# Inactivation of Prefrontal Cortex Delays Emergence From Sevoflurane Anesthesia

**DOI:** 10.3389/fnsys.2021.690717

**Published:** 2021-07-09

**Authors:** Emma R. Huels, Trent Groenhout, Christopher W. Fields, Tiecheng Liu, George A. Mashour, Dinesh Pal

**Affiliations:** ^1^Department of Anesthesiology, University of Michigan, Ann Arbor, MI, United States; ^2^Neuroscience Graduate Program, University of Michigan, Ann Arbor, MI, United States; ^3^Center for Consciousness Science, University of Michigan, Ann Arbor, MI, United States

**Keywords:** anesthesia, consciousness, parietal cortex, prefrontal cortex, rat, righting reflex, sevoflurane

## Abstract

Studies aimed at investigating brain regions involved in arousal state control have been traditionally limited to subcortical structures. In the current study, we tested the hypothesis that inactivation of prefrontal cortex, but not two subregions within parietal cortex—somatosensory barrel field and medial/lateral parietal association cortex—would suppress arousal, as measured by an increase in anesthetic sensitivity. Male and female Sprague Dawley rats were surgically prepared for recording electroencephalogram and bilateral infusion into prefrontal cortex (*N* = 13), somatosensory barrel field (N = 10), or medial/lateral parietal association cortex (*N* = 9). After at least 10 days of post-surgical recovery, 156 μM tetrodotoxin or saline was microinjected into one of the cortical sites. Ninety minutes after injection, rats were anesthetized with 2.5% sevoflurane and the time to loss of righting reflex, a surrogate for loss of consciousness, was measured. Sevoflurane was stopped after 45 min and the time to return of righting reflex, a surrogate for return of consciousness, was measured. Tetrodotoxin-mediated inactivation of all three cortical sites decreased (*p* < 0.05) the time to loss of righting reflex. By contrast, only inactivation of prefrontal cortex, but not somatosensory barrel field or medial/lateral parietal association cortex, increased (*p* < 0.001) the time to return of righting reflex. Burst suppression ratio was not altered following inactivation of any of the cortical sites, suggesting that there was no global effect due to pharmacologic lesion. These findings demonstrate that prefrontal cortex plays a causal role in emergence from anesthesia and behavioral arousal.

## Introduction

Studies over the past century have demonstrated an unequivocal role for subcortical nuclei in behavioral arousal. In particular, lesions in brainstem and the adjoining basal forebrain have been shown to disrupt consciousness and produce a coma-like state or behavioral unresponsiveness (Lindsley et al., 1950; Parvizi and Damasio, 2003; Fuller et al., 2011; Hindman et al., 2018), whereas stimulation of structures within these areas was shown to produce behavioral arousal (Moruzzi and Magoun, 1949; Solt et al., 2014; Vazey and Aston-Jones, 2014; Muindi et al., 2016; Taylor et al., 2016; Luo et al., 2018; Gao et al., 2019; Wang et al., 2019). Of note, these subcortical regions send projections to cortex (Briand et al., 2007; Hoover and Vertes, 2007) but there is limited evidence of a direct role of cortical areas in regulating behavioral arousal. The cortical mechanisms of consciousness have been a subject of recent debate, with specific controversy as to whether the prefrontal cortex plays a causal role (Boly et al., 2017; Odegaard et al., 2017; Raccah et al., 2021). We recently demonstrated that cholinergic stimulation of prefrontal cortex—via local carbachol delivery—in anesthetized rats induced wakefulness despite the continued presence of sevoflurane anesthesia (Pal et al., 2018). In contrast, cholinergic stimulation of parietal cortex in sevoflurane-anesthetized rats failed to produce behavioral arousal (Pal et al., 2018). We also demonstrated that infusion of carbachol into prefrontal cortex during slow-wave sleep reduced the latency to the onset of wakefulness and increased the time spent in wakefulness (Parkar et al., 2020). These studies suggest a causal role for prefrontal cortex in behavioral arousal. However, there is a lack of evidence supporting the necessity of intact prefrontal cortex for arousal and there has been no causal study to investigate the comparative roles of prefrontal and parietal cortices in anesthetic state transitions. Therefore, we used anesthetic state transitions as a model system to systematically compare the effect of pharmacologic inactivation of (1) prefrontal cortex, and (2) two subregions within parietal cortex—somatosensory barrel field (S1BF) and medial/lateral parietal association cortex (M/LPtA) —on behavioral arousal. Based on our recent studies (Pal et al., 2018; Parkar et al., 2020), we hypothesized that inactivation of prefrontal, but not parietal, cortex would facilitate anesthetic induction and delay the emergence from sevoflurane anesthesia. To test the hypothesis, we implemented a loss-of-function approach using tetrodotoxin (TTX)-mediated inactivation of prefrontal or parietal cortices prior to sevoflurane anesthesia and measured the time to loss and return of righting reflex—surrogates for, respectively, loss and return of consciousness in rodents. We demonstrate that, while inactivation of all three cortical sites reduced the time to loss of righting reflex after sevoflurane anesthesia, only prefrontal cortex inactivation increased the time to return of righting reflex, i.e., delayed the emergence time from sevoflurane anesthesia.

## Materials and Methods

The experimental procedures were approved by the Institutional Animal Care and Use Committee at the University of Michigan and were conducted in compliance with the Guide for the Care and Use of Laboratory Animals (National Academies Press, 8th Edition, Washington DC, 2011). The experiments were conducted on adult Sprague Dawley rats (300–350 g, Charles River Laboratories Inc., MA) of both sexes (male = 17, female = 15). The rats were singly housed in a temperature-controlled facility, provided with *ad libitum* food and water, and maintained on a 12 h:12 h light:dark cycle (lights ON at 8:00 am).

### Surgical Procedures

For surgical implantation, rats were placed in an air-tight clear rectangular chamber (10.0 inches × 4.8 inches × 4.2 inches) to induce general anesthesia with 4–5% isoflurane (Piramal Enterprises, Telangana, India) in 100% oxygen. The cranial surface between the eyes and neck was shaved and the rats were positioned in a stereotaxic frame (Model 963, David Kopf Instruments, Tujunga, CA) using blunt ear bars. Isoflurane during surgery was delivered via a rat nose cone (Model 906, David Kopf Instruments, Tujunga, CA) mounted on the stereotaxic frame and was titrated (1–2%) to maintain general anesthesia. The rats were monitored throughout the surgery (at least every 15 min) to ensure the absence of pedal withdrawal reflex, presence of regular breathing pattern, and that the color of extremities stayed pink with a capillary refill time of less than 2 s. The anesthetic concentration was continuously monitored using an anesthetic agent analyzer (Datex Medical Instrumentation, Tewksbury, MA). The body temperature was monitored using a rectal probe (RET-2 ISO, Physitemp Instruments, Inc., Clifton, NJ) and maintained at 37.0 ± 1°C using a small animal far-infrared heating pad (Kent Scientific Co., Torrington, Connecticut). Under aseptic conditions, the cranium was exposed and stainless-steel screw electrodes were implanted across frontal, parietal, and occipital cortices to record electroencephalogram (EEG). A stainless-steel electrode was implanted over the nasal sinus to serve as a reference electrode. Thereafter, the rats were divided into three groups for implantation of bilateral guide cannulae (26G, P1 Technologies, Roanoke, VA) aimed at the following cortical sites: (1) prefrontal cortex, *N* = 13; from Bregma: anterior 3.0 mm, mediolateral 0.7 mm, ventral 3.0 mm, (2) S1BF, *N* = 10; from Bregma: posterior 3.48 mm, mediolateral 5.5 mm, ventral 2.0 mm, and (3) M/LPtA, *N* = 9; from Bregma: posterior 3.72 mm, mediolateral 3.0 mm, ventral 0.5 mm. The areas S1BF and M/LPtA were selected as parietal subregions due to their distinct functional specializations; S1BF is a sensory area while M/LPtA is involved in attention. The free ends of the EEG electrodes were soldered into an electronic connector that, along with the guide cannulae, were affixed on the cranium using dental acrylic (Cat No. 51459, Stoelting Co, Woodlake, IL). The rats received carprofen (5 mg/kg, s.c.) and buprenorphine (0.01 mg/kg, s.c., Buprenex, Reckitt Benckiser Pharmaceuticals, Richmond, VA) for pre-emptive pre-surgical analgesia, and cefazolin (West-Ward-Pharmaceutical, Eatontown, NJ) (20 mg/kg, s.c.) as pre-surgical antibiotic. The rats received buprenorphine (0.03 mg/kg, s.c.) every 8–12 h for 48 h for post-surgical analgesia.

### Experimental Design

**FIGURE 1:**
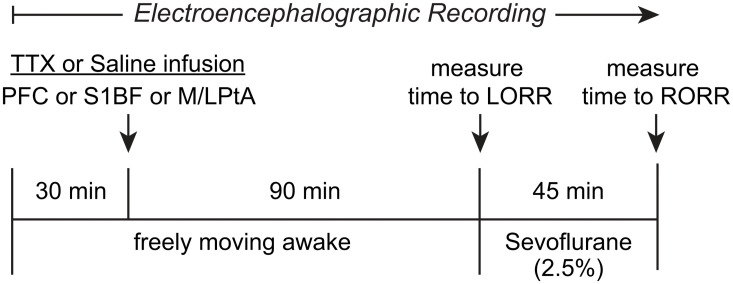
Schematic illustrating the experimental design and timeline. After a baseline wake period of 30 min, tetrodotoxin (TTX) or saline were injected bilaterally into prefrontal cortex (PFC), somatosensory barrel field (S1BF), or medial/lateral parietal association cortex (M/LPtA). Ninety minutes after the TTX injection, sevoflurane administration (2.5%) was started and the time to loss of righting reflex (LORR) was measured. Sevoflurane anesthesia was stopped after 45 min and the time to return of righting reflex (RORR) was measured. The EEG data were collected for the entire duration of the experiment.

The experimental design and timeline are illustrated in Figure 1. The rats were provided at least 10 days of post-surgical recovery period during which they were habituated to the experimental set-up and the EEG recording cable. On the day of the experiment, the rats were connected to the EEG recording system (Blackrock Microsystems, Salt Lake City, UT) between 9:00 and 11:00 a.m. After 30–60 min of the conditioning period to minimize the handling effects, EEG was recorded for 30 min while the rats were kept awake via gentle tapping on the recording chamber and introduction of novel stimuli to maintain a consistent state of arousal. Thereafter, injector cannulae connected via PE tubing to Hamilton syringes mounted on an automatic syringe pump (WPI Inc.) were lowered into the target areas for bilateral microinjection (500 nL at a rate of 100 nL/min) of either 0.9% sterile saline or 156 μM TTX. The injector cannulae protruded 1.0 mm beyond the guide cannulae. The concentration of TTX was based on previously published studies (Sanford et al., 2005; Tang et al., 2005). After TTX or saline injection, EEG data were collected for 90 min while the rats were kept awake using gentle tapping on the recording chamber and/or introduction of novel stimuli. Thereafter, the rats were transferred to an air-tight rectangular chamber (10.0 inches × 4.8 inches × 4.2 inches) for anesthetic induction with 2.5% sevoflurane (2 L/min) in 100% oxygen. To assess loss of righting reflex (LORR), a surrogate for loss of consciousness in rodents, the chamber was slowly rotated by the experimenter to place the rat in a supine position. LORR was determined by the inability of the rat, once placed in a supine position, to right itself on all four paws within 30 s. At the onset of LORR, the rat was placed in a supine position in a custom-built plexiglass clear cylindrical chamber (10–11 L) and was connected to the EEG recording cable. A rectal probe (RET-2 ISO, Physitemp Instruments, Inc., Clifton, NJ) was positioned and was connected via a feedback temperature controller (TCAT-2LV, Physitemp Instruments, Inc., Clifton, New Jersey) to a small animal heating pad (Kent Scientific Co., Torrington, Connecticut) to maintain the core body temperature at 37 ± 1°C. A pulse oximetry sensor (MouseOx, Starr Life Science Corp., Oakmont, PA) was positioned on the hind paw or around the neck to monitor heart and respiration rate, and oxygen saturation. General anesthesia was maintained with 2.5% sevoflurane (10 L/min) for 45 min while the sevoflurane concentrations at the gas inlet and output ports of the recording chamber were continuously monitored using two anesthesia monitors (Fukuda Denshi United States, INC., Redmond, WA). At the completion of 45 min, sevoflurane administration was stopped and the time to return of righting reflex (RORR), a surrogate for return of consciousness in rodents, was measured. RORR was determined as the time point at which the rat was able to right itself from the supine position to upright posture on all four paws. Given the subjective nature of LORR/RORR determination, and to confirm the reproducibility of the effect of TTX injection on the changes in the time to LORR and RORR within our experimental paradigm, we repeated the TTX injection session in the entire prefrontal cohort (*N* = 13) and 7 out of 10 rats in S1BF cohort i.e., each rat received one saline (vehicle control) and two TTX injections separated by at least 5–7 days. The injections, separated by 5–7 days, were done in a randomized counter-balanced manner in which some animals received saline first followed by two consecutive TTX sessions, some animals received two consecutive sessions of TTX followed by a saline session, and some of the animals received a TTX session, followed by a saline session and then the repeat of the TTX session. Statistical analysis showed no significant difference (see “Results” section) in the time to LORR or RORR between the two TTX sessions in the prefrontal cortex and S1BF cohorts. Therefore, to minimize the exposure to this biohazard, we performed only one TTX session for the M/LPtA cohort.

### Electroencephalographic Recordings

The EEG data were acquired using a Cereplex μ headstage connected to a CerePlex Direct recording system (Blackrock Microsystems LLC, Salt Lake City, UT). Monopolar frontal and parietal EEG (0.1–500 Hz, 1 kHz sampling rate) were used for burst suppression analysis.

### EEG Burst Suppression Analysis

Burst suppression ratio was computed using methods previously described in a study from our laboratory (Hambrecht-Wiedbusch et al., 2017). The EEG data were bandpass filtered between 5 and 30 Hz using a 4-order Butterworth filter with a zero-phase forward and reverse algorithm. To estimate the instantaneous amplitude of the signal, the EEG data were Hilbert transformed (Lewis et al., 2013) and then further smoothed with a moving average filter of 200 ms. Next, a threshold for suppression was computed from a manually labeled suppression period based on visual inspection (mean ± 3–4 standard deviations), which was used to create a binary signal of burst and suppression periods with the minimum length of burst or suppression being 500 ms. The burst suppression ratio (BSR) was calculated as the percentage of time spent in suppression during each minute of EEG data. The BSR was then averaged for the entire 45 min of sevoflurane anesthesia. To ensure that we did not include EEG electrodes that may have been impacted by local injection of TTX, we used parietal EEG for calculation of burst suppression in the prefrontal inactivation group and frontal EEG for the S1BF and M/LPtA inactivation groups. Two rats in the prefrontal cohort were excluded from BSR analysis for a lack of burst suppression across all experiments.

### Histological Verification of the Site of Microinjections

**FIGURE 2:**
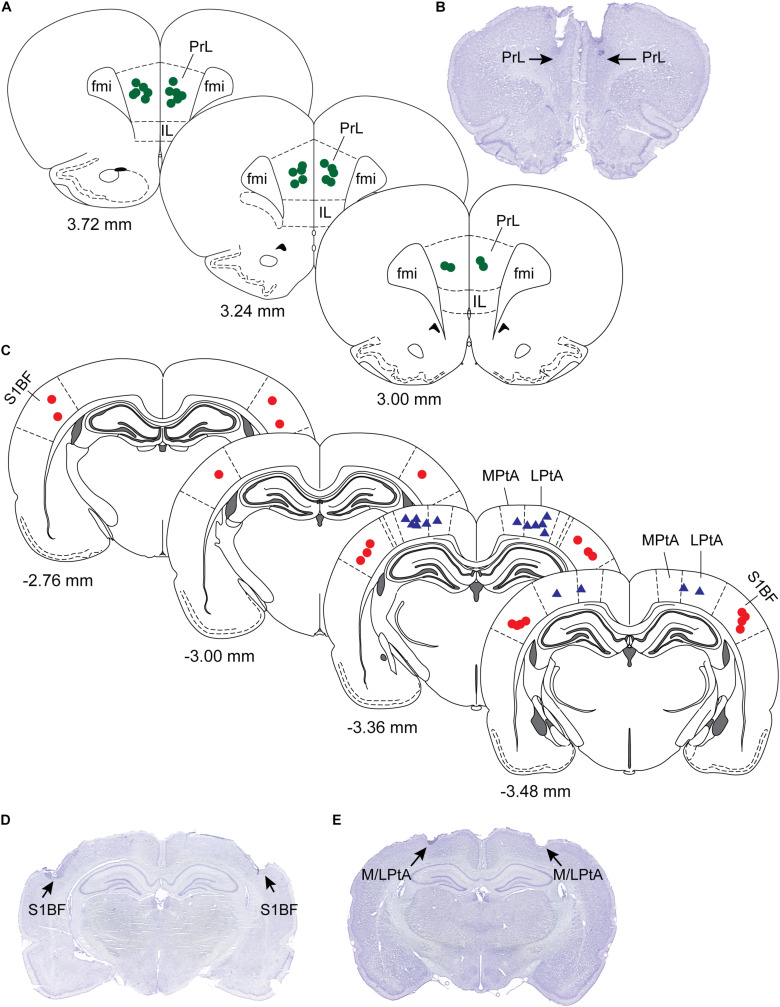
Histological verification of sites of microinjection. The sites of microinjections were confirmed in cresyl violet stained coronal brain sections (40 μm) through prefrontal cortex, somatosensory barrel field (S1BF), and medial/lateral parietal association cortex (M/LPtA). **(A)** Shows microinjection locations (green dots) plotted onto stereotaxic diagrams through prelimbic region (PrL) of the prefrontal cortex, and a representative PrL section is shown in **(B)**. **(C)** Shows the microinjection locations in S1BF (red dots) and medial (MPtA) and lateral parietal association cortex (LPtA) (blue triangles). Representative brain sections for S1BF and M/LPtA are shown in **(D)** and **(E)**, respectively. Each symbol (dots or triangles) represents one rat. The numbers below the stereotaxic diagram are the anteroposterior distance from Bregma. Positive numbers show distance anterior to Bregma while the negative numbers show distance posterior to Bregma. The stereotaxic diagrams are modified from the atlas (The Rat Brain in Stereotaxic Coordinates) by Paxinos and Watson (2007). Fmi; forceps minor of the corpus callosum; IL, infralimbic cortex.

After the completion of all experimental sessions, the rats were euthanized with an overdose of carbon dioxide and perfused intracardially, first with 150 mL of wash solution containing 0.1 M (pH 7.2) phosphate buffered saline (1219SK, EM Sciences, Hatfield, PA) followed by 200 mL of a fixative solution containing 4% paraformaldehyde and 4% sucrose in 0.1 M (pH 7.2) phosphate buffer (1224SK, EM Sciences, Hatfield, PA). The brains were extracted and allowed to equilibrate in 30% sucrose before cryosectioning into 40 μm thick coronal brain sections through prefrontal cortex, S1BF, and M/LPtA. The sections were mounted on glass slides and stained with 2% cresyl violet solution for verification of the site of microinjection (Figure 2).

### Statistical Analysis

The statistical analyses were conducted using R software version 4.0 (R Core Team, 2020) and in consultation with the *Consulting for Statistics, Computing, and Analytics Research* core at the University of Michigan. GraphPad Prism software version 9.1 (GraphPad Software, San Diego, CA, United States) was used to create all graphs. The sample sizes were based on previous studies from our laboratory (Pal et al., 2015, 2016, 2018). A linear mixed model fit with lme4 (Bates et al., 2015) was used for statistical (within-rat) comparison of the time to LORR, time to RORR, and BSR between TTX and saline conditions for each of the three rat cohorts: prefrontal cortex, S1BF, and M/LPtA. The linear mixed model included sex, condition (saline, TTX1, and TTX2), and experimental order of TTX or saline injection as fixed effects and the subject (rat) as a random intercept. Our initial analysis using this model did not reveal any order effect (i.e., order of TTX and saline injections), and therefore, order was subsequently dropped from the statistical model. Tukey’s *post hoc* tests did not show any statistical differences between the effect of first and second TTX session on the time to LORR or RORR. Therefore, we derived a single estimate of the common statistical effect (inferential, not descriptive) of TTX (vs. saline). Similarly, all data points from TTX1 and TTX2 sessions were plotted as a pooled TTX group. The data plots showing the time to LORR and RORR following individual TTX sessions (i.e., TTX1 and TTX2) for the prefrontal cortex and S1BF cohorts are provided in Supplementary Figures 1, 2. Statistical outputs regarding the effect of individual TTX treatments (TTX1 and TTX2) on the time to LORR and RORR are provided in Supplementary Tables 1, 2. The comparisons were considered statistically significant if *p* < 0.05. Data are reported as mean ± standard deviation (SD), followed by t-statistic, *p*-value, 95% confidence interval (CI), and unstandardized beta (β) coefficient.

## Results

Histological analysis confirmed the microinjection sites to be located within the target regions, i.e., prefrontal cortex, S1BF, and M/LPtA (Figure 2).

### TTX-Mediated Inactivation of Prefrontal and Parietal Cortices Accelerated Induction of Anesthesia

There was no significant difference in the time to LORR between the first and second TTX sessions in the prefrontal cortex group [*t*(12) = 1.27, *p* = 0.4] or the S1BF group [*t*(6) = −1.34, *p* = 0.4]. In addition, there was no significant main effect of sex on the time to LORR in any of the three target areas [for prefrontal cortex, mean ± SD: 139.6 s ± 52 for males vs. 127.1 s ± 16.7 for females, *t*(12) = 1.017, *p* = 0.3, 95% CI (−10.89, 35.92), β = 12.51; for S1BF, mean ± SD: 239.9 s ± 135.2 for males vs. 211.5 s ± 47.03 for females, *t*(9) = 0.31, *p* = 0.8, 95% CI (−86.46, 116.65), β = 16.11; for M/LPtA, mean ± SD: 159.6 s ± 40.9 for males vs. 186.9 s ± 44.9 for females, *t*(8) = −1.15, *p* = 0.3, 95% CI (−72.95, 18.50), β = −27.22].

**FIGURE 3:**
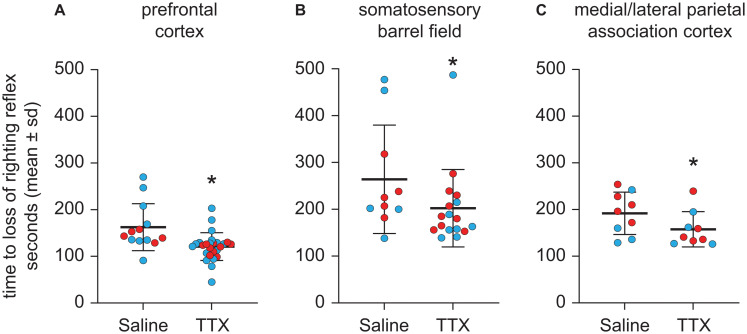
Effect of TTX-mediated inactivation of prefrontal cortex (*N* = 13), somatosensory barrel field (*N* = 10), or medial/lateral parietal association cortex (*N* = 9) on the time to loss of righting reflex after sevoflurane anesthesia. Inactivation of prefrontal cortex **(A)**, somatosensory barrel field **(B)**, and medial/lateral parietal association cortex **(C)** decreased the time to loss of righting reflex after sevoflurane anesthesia. The group data are shown as mean ± standard deviation. Individual rat data are shown as colored dots with red dots representing female rats and blue dots representing male rats. The TTX condition in **(A,B)** show the data points (dots) from both TTX sessions (i.e., TTX1 and TTX2). The significance symbol (*) denotes *p* < 0.05 and shows statistical comparison with the saline injection using a linear mixed model. The actual *p*-values are provided in the text in the results section.

Compared to the time to LORR after saline injection, TTX infusion into prefrontal cortex produced a statistically significant decrease in the time to LORR [mean ± SD: 121 s ± 29.8 for TTX vs. 162.4 s ± 50.3 for saline, *t*(12) = −3.27, *p* = 0.001, 95% CI (−66.35, −16.60), β = −41.48] (Figure 3A). As compared to the time to LORR after saline injection, TTX infusion into S1BF also produced a statistically significant decrease in the time to LORR [mean ± SD: 202.3 s ± 82.6 for TTX vs. 264.1 s ± 115.6 for saline, *t*(9) = −2.53, *p* = 0.01, 95% CI (−129.71, −16.52), β = −73.11] (Figure 3B). Similar to the effect of TTX-mediated inactivation of prefrontal cortex and S1BF, TTX infusion into M/LPtA decreased the time to LORR, as compared to that observed after saline injection [mean ± SD: 157.6 s ± 37.8 for TTX vs. 191.9 s ± 45.4 for saline, *t*(8) = −2.43, *p* = 0.02, 95% CI (−63.40, −5.15), β = −34.28] (Figure 3C).

### TTX-Mediated Inactivation of Prefrontal Cortex, but Not Parietal Cortex, Delayed Emergence From Anesthesia

**FIGURE 4:**
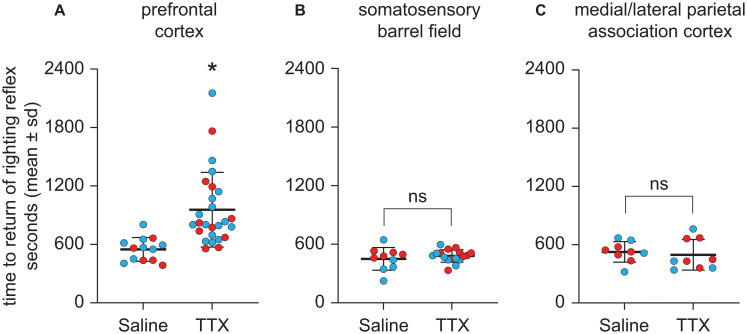
Effect of TTX-mediated inactivation of prefrontal cortex (*N* = 13), somatosensory barrel field (*N* = 10), or medial/lateral parietal association cortex (*N* = 9) on the time to return of righting reflex after sevoflurane anesthesia. Inactivation of prefrontal cortex **(A)** increased the time to return of righting reflex after sevoflurane anesthesia. In contrast, inactivation of neither somatosensory barrel field **(B)** nor medial/lateral parietal association cortex **(C)** had a significant effect on the time to return of righting reflex after sevoflurane anesthesia. The group data are shown as mean ± standard deviation. Individual rat data are shown as colored dots with red dots representing female rats and blue dots representing male rats. The TTX condition in **(A,B)** show the data points (dots) from both TTX sessions (i.e., TTX1 and TTX2). The significance symbol (*) denotes *p* < 0.05 and shows statistical comparison with the saline injection using a linear mixed model. The actual *p*-values are provided in the text in the results section. ns, not significant.

Next, we examined the effect of TTX-mediated inactivation of prefrontal and parietal cortices on the time to RORR (Figure 4). There was no significant difference in the time to RORR between the first and second TTX sessions for the prefrontal cortex group [*t*(12) = 0.72, *p* = 0.8] or the S1BF group [*t*(6) = −0.87, *p* = 0.7]. In addition, there was no significant main effect of sex on time to RORR after TTX infusion into any of the three target areas [for prefrontal cortex, mean ± SD: 846.5 s ± 381.2 for males vs. 778.8 s ± 370.7 for females, *t*(12) = 0.60, *p* = 0.6, 95% CI (−153.19, 288.53), β = 67.67; for S1BF, mean ± SD: 445.5 s ± 107.2 for males vs. 491.3 s ± 56.2 for females, *t*(9) = −1.41, *p* = 0.2, 95% CI (−108.52, 15.26), β = −46.63); for M/LPtA, mean ± SD: 505.5 s ± 169.1 for males vs. 515.2 s ± 101.6 for females, *t*(8) = −0.11, *p* = 0.9, 95% CI (−174.35, 155.055), β = −9.65].

As compared to the time to RORR after saline injection, TTX infusion into prefrontal cortex produced a statistically significant increase in the time to RORR [mean ± SD: 956.6 s ± 383.9 for TTX vs. 548.2 s ± 121.8 for saline, *t*(12) = 3.76, *p* = 0.0002, 95% CI (195.37, 621.42), β = 408.4] (Figure 4A). Inactivation of S1BF did not produce any statistically significant effect on the time to RORR [mean ± SD: 480.4 s ± 64.6 for TTX vs. 450.2 s ± 115.7 for saline, *t*(9) = 0.75, *p* = 0.5, 95% CI (−41.62, 92.85), β = 25.61] (Figure 4B). Similarly, inactivation of M/LPtA did not produce any statistically significant effect on the time to RORR [mean ± SD: 496.2 s ± 158.3 for TTX vs. 525.6 s ± 105.6 for saline, *t*(8) = −0.70, *p* = 0.5, 95% CI (−115.52, 56.74), β = −29.39] (Figure 4C).

### TTX-Mediated Inactivation of Prefrontal or Parietal Cortex Did Not Affect Burst Suppression Ratio

**FIGURE 5:**
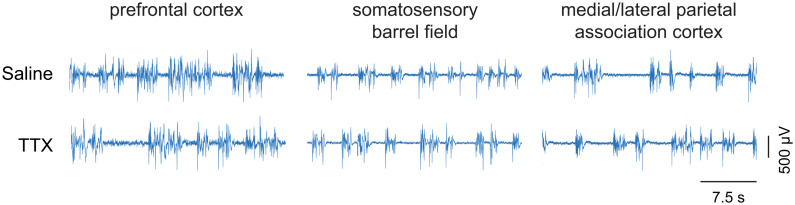
Representative EEG traces showing burst suppression pattern. The EEG traces show the pattern of burst suppression after saline and tetrodotoxin (TTX) microinjection into prefrontal cortex, somatosensory barrel field, and medial/lateral parietal association cortex. The horizontal bar on the lower right is the scale for time. The vertical bar on the lower right is the EEG amplitude scale.

**FIGURE 6:**
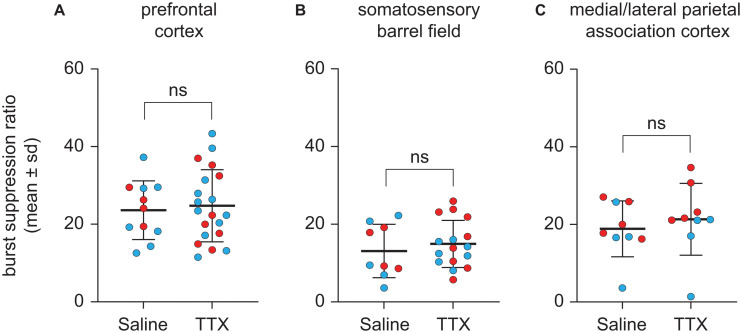
Effect of TTX-mediation inactivation of prefrontal cortex (*N* = 13), somatosensory barrel field (*N* = 10), or medial/lateral parietal association cortex (*N* = 9) on burst suppression ratio during sevoflurane anesthesia. Statistical comparisons using a linear mixed model showed that as compared to saline controls, inactivation of prefrontal cortex **(A)**, somatosensory barrel field **(B)** or medial/lateral parietal association cortex **(C)** did not affect the burst suppression ratio during sevoflurane anesthesia. Two rats in the prefrontal cohort were excluded from analysis for a lack of burst suppression across all experiments. The group data are shown as mean ± standard deviation. Individual rat data are shown as colored dots with red dots representing female rats and blue dots representing male rats. The TTX condition in **(A,B)** show the data points (dots) from both TTX sessions (i.e., TTX1 and TTX2). ns, not significant.

Representative EEG traces showing burst suppression pattern after microinjection of saline or TTX are shown in Figure 5. There were no sex-related differences in the burst suppression ratio (BSR) after TTX infusion into any of the three cortical sites [for prefrontal cortex, mean ± SD: 27.4 ± 8.4 for males vs. 34.1 ± 12.4 for females, *t*(10) = −1.35, *p* = 0.2, 95% CI (−17.61, 3.075), β = −7.22; For S1BF, mean ± SD: 15.8 ± 6.9 for males vs. 19.7 ± 8.5 for females, *t*(9) = −0.9, *p* = 0.4, 95% CI(−11.2, 4.1), β = −3.59; for M/LPtA, mean ± SD: 19.3 ± 10.7 for males vs. 29.8 ± 7.2 for females, *t*(8) = −1.91, *p* = 0.06, 95% CI(−21.07, 0.13), β = −10.47]. As compared to BSR after saline injection, there was no statistical change in BSR after TTX infusion into any of the cortical sites [for prefrontal cortex, mean ± SD: 31 ± 11.6 for TTX vs. 29.5 ± 9.4 for saline, *t*(10) = 0.26, *p* = 0.8, 95% CI(−4.59, 6.024), β = 0.72; for S1BF, mean ± SD: 18.7 ± 7.6 for TTX vs. 16.4 ± 8.6 for saline, *t*(9) = 0.7, *p* = 0.5, 95% CI (−3.55, 7.46), β = 1.96; for M/LPtA, mean ± SD: 26.7 ± 11.6 for TTX vs. 23.6 ± 9 for saline, *t*(8) = 1.08, *p* = 0.3, 95% CI(−2.81, 8.99), β = 3.09] (Figure 6).

## Discussion

In this study, we demonstrate that inactivation of prefrontal cortex, S1BF, and M/LPtA facilitated entry into the anesthetized state whereas only the inactivation of prefrontal cortex impaired the exit from the anesthetized state. This supports a role for the prefrontal cortex as an important node in arousal circuitry and modulator of consciousness.

It is important to note that these three cortical sites have distinct functional specializations. The prefrontal cortex is associated with cognitive control (Miller and Cohen, 2001; Widge et al., 2019), S1BF is a sensory region (Borich et al., 2015), and M/LPtA is implicated in attention and sensory integration (Behrmann et al., 2004; Reep and Corwin, 2009). Despite these functional specializations, all three sites appear to regulate the transition to anesthetic-induced unconsciousness whereas only the prefrontal cortex appeared to influence emergence from anesthesia. A role for prefrontal cortex in emergence from unconscious states is also supported by studies from our and other laboratories. As noted, we recently demonstrated that cholinergic stimulation of prefrontal cortex, but neither S1BF nor MPtA, induced wakefulness in sevoflurane-anesthetized rats (Pal et al., 2018). We also showed that cholinergic stimulation of prefrontal cortex during slow-wave sleep decreased the latency to the onset of wakefulness and increased the total time spent in wake state (Parkar et al., 2020). A rat study focused on thalamocortical synchronization during anesthesia demonstrated similar neural dynamics between rat and human prefrontal cortex and posited a role for prefrontal cortex in emergence from anesthesia (Flores et al., 2017). Furthermore, studies in human patients also support a role for prefrontal cortex in recovery of consciousness: transcranial direct current stimulation of prefrontal cortex led to improved outcomes in patients in a minimally conscious state (Angelakis et al., 2014; Thibaut et al., 2014, 2017), and increased thalamocortical connectivity to prefrontal cortex was associated with recovery from vegetative state (Jang et al., 2020). Application of transcranial direct current stimulation to frontal motor cortex of rats was also reported to accelerate emergence from isoflurane anesthesia (Mansouri and García, 2021). This role for prefrontal cortex in modulating level of consciousness could be predicted based on the rich interconnectivity of prefrontal cortex and various arousal-promoting nuclei in the brainstem and diencephalon (Briand et al., 2007; Hoover and Vertes, 2007).

Parietal cortex contains multiple sensory (e.g., S1BF) and association (e.g., M/LPtA) regions that are implicated in stimulus integration (Behrmann et al., 2004; Reep and Corwin, 2009) and has been thought to be crucial for the contents of consciousness, i.e., particular qualities of subjective experience (Koch et al., 2016a,b; Boly et al., 2017). Our data suggest that parietal regions may play a permissive role during transitions from consciousness to unconsciousness. Specifically, suppression of activity in regions responsible for processing sensory stimulation and/or attention may facilitate anesthetic-induced loss of consciousness. Furthermore, lack of effect of inactivation of S1BF or M/LPtA on emergence from sevoflurane anesthesia is also supported by our previous work in which we demonstrated that cholinergic stimulation of S1BF and MPtA did not facilitate emergence from sevoflurane anesthesia (Pal et al., 2018). The differential role of these cortical sites in anesthetic induction and emergence supports the hypothesis that anesthetic induction and emergence are not mirror images of each other but rather are regulated by distinct neural processes (Friedman et al., 2010; Tarnal et al., 2016).

The current study also informs the interpretation of anesthetic recovery studies in healthy humans. For example, carefully designed positron emission tomography studies in humans have not identified prefrontal cortex or frontal-parietal networks as playing a prominent role in the recovery of consciousness in the setting of sedative or anesthetic exposure (Xie et al., 2011; Långsjö et al., 2012; Scheinin et al., 2020). However, even these experimentally sophisticated protocols are correlational in nature and therefore do not form a firm basis for conclusions regarding the specific role of cortical sites in recovery of consciousness. By contrast, our loss-of-function design enables causal inference regarding the role of prefrontal and posterior parietal cortices in anesthetic state transitions. Furthermore, the current data align with a multicenter study of healthy human volunteers finding that recovery of prefrontal electroencephalographic dynamics, which occur on a finer temporal scale than positron emission tomography, preceded return of consciousness and appeared more active than posterior cortex (Mashour et al., 2021). These data support the possibility of the translational relevance of our findings in rodents, but further investigation is clearly warranted.

Of note, our results show that TTX-mediated inactivation of the cortical sites (prefrontal cortex, S1BF, M/LPtA) did not affect the level of burst suppression, as compared to saline controls, during continuous sevoflurane anesthesia. This suggests that the increase in anesthetic potency after TTX-mediated inactivation of cortical sites was due to focal changes (i.e., inactivation of specific cortical sites) rather than a generalized dampening of neural activity across the brain.

One major limitation of using TTX for pharmacological lesions is that it produces non-specific neuronal inactivation along with inactivation of neuronal fibers (Martin and Ghez, 1999), which precludes us from commenting on either the phenotype of neurons that mediate these changes in behavioral arousal or the role that fibers of passage may play. However, the aim of our study was to understand the role of cortical regions in behavioral arousal rather than specific neuronal subpopulations. Further studies using neuron-specific inhibitors (e.g., muscimol) and probes to target individual neuronal types are required for a more granular analysis of the role of these cortical areas in consciousness. Although we did not directly assess the inactivating effect of TTX on these brain regions, the concentration of TTX used in the current study was based on previous studies that demonstrated quantifiable effect on behavior (Sanford et al., 2005; Tang et al., 2005) as well as direct inhibitory effect on neuronal activity (van Duuren et al., 2007). We studied the anesthetic state transitions at one fixed sevoflurane and TTX concentration. The choice of using sevoflurane at ∼1 MAC was guided by our aim to maintain a stable plane of anesthesia, which would have been difficult at a lower concentration. Similarly, lower doses of TTX could have resulted in partial or incomplete inactivation of neuronal populations, which would have increased the likelihood of higher variability in behavioral response and hence difficulty in interpreting the results.

Unlike the quantification of return of righting reflex, which is measured against the backdrop of a stable plane of anesthesia and quiescent behavior, measurement of time to loss of righting reflex is inherently variable. The process to determine loss of righting reflex in rodents involves repeatedly positioning them in the supine posture, which is a stimulus that naturally interferes with anesthetic induction and thus can artificially prolong the process in an unpredictable way, increasing the variability in the data. The rodents also show a strong resistance to loss of consciousness, which is perhaps evolutionarily associated with avoiding a highly vulnerable supine position. Therefore, results from loss of righting reflex experiments should be interpreted with caution. Despite these limitations, the loss of righting reflex (along with the return of righting reflex) is a well-established and widely used surrogate for anesthetic potency that has yielded critical insights into mechanisms of anesthesia and consciousness (McCarren et al., 2013; Leung et al., 2021). Of note, we did not measure the spatial spread of TTX in our study and thus cannot exclude the possibility that the areas adjoining prefrontal cortex, S1BF, or M/LPtA could have been affected. However, the prefrontal cortex and S1BF have an anteroposterior expanse of more than 2mm, and our histological analysis found the injection sites to be within these regions. M/LPtA also has an anteroposterior expanse of about 1 mm and our injection sites were located within this target region. Furthermore, a volume of 500 nL (as was administered in our study) is not expected to have a spread of more than 1–1.5 mm and the concentration of TTX is expected to progressively dilute as it spreads outwards from the injection site. Given these considerations, it is possible but unlikely that the areas outside the target regions would have been affected in our study. Even in the eventuality of TTX affecting the neural activity in adjoining areas, our primary finding that frontal cortex (as opposed to parietal cortex) has a causal influence on behavioral arousal and emergence from anesthesia is not mitigated.

## Conclusion

We employed a loss-of-function approach and used anesthetic state transitions as a model system to determine the role of prefrontal and parietal cortices in consciousness. Our findings suggest that prefrontal cortex, but not parietal cortex, plays a causal role in behavioral arousal and provide further direct evidence that the prefrontal cortex is a critical node in the arousal circuitry that controls consciousness.

## Data Availability Statement

The raw data and analysis scripts utilized in this manuscript can be made available on request to the corresponding author.

## Ethics Statement

The animal study was reviewed and approved by the Institutional Animal Care and Use Committee, University of Michigan.

## Author Contributions

EH designed the study, performed the experiments, analyzed the data, and wrote the manuscript. TG and TL performed the experiments. CF conducted the histological analysis. GM designed the study and wrote the manuscript. DP designed the study, analyzed the data, and wrote the manuscript. All authors contributed to the article and approved the submitted version.

## Conflict of Interest

The authors declare that the research was conducted in the absence of any commercial or financial relationships that could be construed as a potential conflict of interest.
