# Supplementary material for: Inactivation of Prefrontal Cortex Delays Emergence From Sevoflurane Anesthesia
Source: Front Syst Neurosci. 2021 Jul 9;15:690717. doi: 10.3389/fnsys.2021.690717 (PMC8299111; doi:10.3389/fnsys.2021.690717)
Supplement: Supplementary file 3 [file Table_1.docx]

|  | **𝛽** | **95% CI** | ***p value*** |
| --- | --- | --- | --- |
| Prefrontal Cortex |  |  |  |
| Condition: TTX1 vs. Saline | -50.8 | [-78.7, -22.9] | 0.002 |
| Condition: TTX2 vs. Saline | -32.2 | [-60.05, -4.3] | 0.07 |
| S1BF |  |  |  |
| Condition: TTX1 vs. Saline | -49.0 | [-111.3, 13.3] | 0.3 |
| Condition: TTX2 vs. Saline | -97.2 | [-165.3, -22.7] | 0.02 |

**Supplementary Table 1.** Statistical output for the effect of TTX1 and TTX2 sessions on the time (seconds) to loss of righting reflex

𝛽 = unstandardized coefficient; CI = confidence interval [lower, upper]
