# Supplementary material for: Inactivation of Prefrontal Cortex Delays Emergence From Sevoflurane Anesthesia
Source: Front Syst Neurosci. 2021 Jul 9;15:690717. doi: 10.3389/fnsys.2021.690717 (PMC8299111; doi:10.3389/fnsys.2021.690717)
Supplement: Supplementary file 4 [file Table_2.docx]

**Supplementary Table 2.** Statistical output for the effect of TTX1 and TTX2 sessions on the time (seconds) to return of righting reflex

|  | **𝛽** | **95% CI** | ***p value*** |
| --- | --- | --- | --- |
| Prefrontal Cortex |  |  |  |
| Condition: TTX1 vs. Saline | 363.2 | [118.9, 607.5] | 0.01 |
| Condition: TTX2 vs. Saline | 453.6 | [209.3, 697.9] | <0.001 |
| S1BF |  |  |  |
| Condition: TTX1 vs. Saline | 43.9 | [-27.8, 115.6] | 0.5 |
| Condition: TTX2 vs. Saline | 7.3 | [-71.8, 86.5] | 1.0 |

𝛽 = unstandardized coefficient; CI = confidence interval [lower, upper]
